# Understanding the feasibility of yoga and compassion meditation for stress management in mothers of children with intellectual disabilities in Nepal: a qualitative exploration

**DOI:** 10.1080/16549716.2025.2582260

**Published:** 2025-11-11

**Authors:** Namrata Pradhan, Gørill Haugan, Poonam Rishal, Jennifer Infanti

**Affiliations:** a Faculty of Medicine and Health Sciences, Department of Public Health and Nursing, Norwegian University of Science and Technology (NTNU), Trondheim, Norway; b Faculty of Nursing and Health Sciences, Nord University, Levanger, Norway; c Department of Community Medicine, Kathmandu Medical College Public Limited, Kathmandu University, Kathmandu, Nepal

**Keywords:** caregiver, maternal wellbeing, unpaid carer, mindfulness-based intervention, contextual adaptation, low-income country, South Asia

## Abstract

**Background:**

Mothers caring for children with intellectual disabilities often experience high levels of stress and mental health challenges, underscoring the need for targeted health-promoting interventions.

**Objective:**

This qualitative study explores the feasibility of brief yoga and compassion meditation training for stress management among mothers of children with intellectual disabilities in Nepal, focusing on their experiences and insights.

**Methods:**

Twenty-four mothers participated in three separate focus group discussions (FGDs) each with a different group of participants and held in different locations, after completing a yoga and compassion meditation program (YCMP). Thematic analysis was used to explore the intervention’s feasibility, based on participants’ perception of its acceptability, practicality, appropriateness, adaptability, and implementation demands, along with challenges to address before broader application in health-promotion efforts.

**Results:**

Participants reported that the YCMP improved their emotional and physical wellbeing, was easily integrated into daily routines, was culturally and contextually appropriate, and met their desire for non-invasive interventions. The FGDs also revealed demands for such interventions. However, some participants noted challenges, notably time constraints, which limit their ability to engage in regular yoga and meditation practice.

**Conclusions:**

The YCMP was valued as a culturally and contextually appropriate approach to managing caregiver stress. Adaptability, low cost, and non-invasive nature supports further research and potential implementation. In addition, insights from the FGDs provide guidance for the ongoing customization and expansion of the intervention.

## Paper Context

● **Main findings:** A Yoga and Compassion Meditation Program (YCMP) improved wellbeing, was culturally appropriate, and practical for mothers of children with intellectual disabilities in Nepal, though time constraints limited its use.

● **Added knowledge:** Demonstrates that the YCMP is feasible and acceptable in a low-resource cultural context, with time constraints as a key barrier.

● **Global health impact for policy and action:** Supports integrating low-cost, culturally-adapted stress management interventions into health systems in resource-limited settings.

## Background

Globally, approximately 52.9 million children under the age of five have some form of developmental disability, with nearly 95% living in low- and middle-income countries (LMICs) [1]. Intellectual disability (ID), which has remained the leading contributor to years lived with disability since 1990, is particularly prevalent in South Asia, where it imposes substantial burdens, especially in resource-poor settings [1]. ID impacts not only the lives of affected children but also their family members, who often face sustained emotional, social, and economic challenges due to the ongoing demands of caregiving. According to the Diagnostic and Statistical Manual of Mental Disorders, fifth edition (DSM-5), ID involves significant limitations in both intellectual functioning (such as reasoning, problem-solving, and learning) and adaptive behaviour, which includes conceptual, social, and practical skills [2]. Similarly, the American Association on Intellectual and Developmental Disabilities (AAIDD) defines ID as originating before the age of 18 and emphasises the interaction between the individual and their environment, highlighting the role of tailored support in improving functioning [3]. In this study, we adopt a broad view of ID that considers both medical and social factors, including neurodevelopmental differences and the influence of context and structures, rather than relying solely on a medical model. Children with ID, especially those with associated medical comorbidities, often require more intensive and ongoing care than typically developing children. This care is mostly provided by parents or family members, which can significantly impact caregivers’ emotional and physical wellbeing, quality of life, and health [4].

Parental stress and the risk of depression associated with raising and caring for children with ID are well documented [5–7], including our research involving Nepali mothers [8–10]. Caregiver stress levels are influenced by intrinsic factors (e.g. the severity of the disability and challenging behaviours) and extrinsic factors (e.g. family circumstances and social support) [4]. High stress levels negatively impact caregivers’ health and caregiving abilities, thus underscoring the need for health support for both parents and children with ID [5]. Despite high rates of parental depression, this condition is often undiagnosed and untreated, and there is limited availability of counselling services for these parents [11,12].

In many LMICs, direct government-led social welfare or financial assistance for family caregivers of children with ID, who are predominantly mothers, is limited or unavailable [13,14]. Most available interventions are developed or implemented by non-governmental organisations (NGOs), research institutions, or community organisations rather than through sustained public welfare programs [15]. In Nepal, mothers of children with ID shoulder diverse responsibilities beyond caregiving, including household chores, care for extended families, and often paid work, all with minimal external support such as support from government or community services [13]. Consequently, they face significant health challenges, including high levels of stress [9,16]. Our previous research has documented various stressors – emotional, physical, financial, and social – that impact on these mothers’ wellbeing and quality of life and lead to increased perceived stress, anxiety, depression, and isolation [10]. Social stigma and discrimination related to disabilities, including both the individuals with disabilities and their families, further exacerbate these burdens [6,17]. Thus, health-promoting interventions that support caregivers of children with ID are critically needed, as is knowledge about the efficacy of such interventions in LMICs.

To date, research on such strategies to support the mental health of mothers of children with ID in Nepal and other LMICs is scarce. A 2021 systematic review identified only two studies, both from Iran, that explored psychological interventions for improving parental wellbeing, and it identified three studies from India, China, and Vietnam that focused on enhancing parenting skills to support the development of their children with ID [18]. Additionally, a pilot study from India aimed at promoting wellbeing, resilience, and stress management among mothers of children with ID was identified [19]. This scarcity highlights the lack of tailored health promotion interventions for these mothers in LMIC contexts.

Beyond peer-reviewed research, a small but growing body of grey literature, including project reports and postgraduate theses, has explored related themes. For example, Carers Worldwide’s telephone helpline and Carers Association Project provided direct psychological and advocacy support to unpaid caregivers in multiple districts of Nepal [20]. Similarly, a 2020 PhD thesis [21] explored parental caregiving experiences and barriers to education for children with disabilities in Nepal. Such findings, while valuable, are rarely integrated into formal systematic reviews or programmatic guidelines.

Globally, various strategies have been developed to address the stress and wellbeing needs of mothers of children with ID [22], including psychoeducation, mindfulness-based interventions, cognitive-behavioural therapy (CBT), peer support groups, and relaxation techniques [19,23–25]. Emerging grey and programmatic literature includes a thesis on coping strategies of parents of children with developmental delay, which provides empirical parental insights typically missing from the published literature [26] Furthermore, global initiatives such as the World Health Organization’s Caregiver Skills Training (CST) [27] and UNICEF’s Caring for the Caregiver [28] intervention offer replicable models that integrate caregiver wellbeing in broader support systems in LMICs. This pattern highlights that, while practical parenting skills receive considerable emphasis, dedicated wellbeing-focused support for caregivers remains underexplored. Such approaches have been shown to help mothers build positive coping skills, shift negative thought patterns, and gain emotional support through shared experiences and mutual understanding [16,18,20–24].

However, most of these interventions are not culturally relevant or are not accessible to mothers of children with ID in Nepal [18]. It remains unclear which mental health and social interventions for caregivers of children with ID are effective in South Asian contexts [22]. Culturally embedded expressions and understandings of emotional distress in South Asia differ from Western contexts, potentially reducing the effectiveness of interventions like CBT [24,25]. Furthermore, stigma and a shortage of trained mental health professionals complicate the implementation of psychological counselling in Nepal [15,29–31], highlighting the need for simple, low-cost, culturally aligned, and accessible approaches to manage stress.

### Culturally relevant interventions in Nepal

Yoga and meditation are culturally accepted health-promoting practices in Nepal, and they both show evidence of reducing stress and improving health outcomes among mothers of children with ID in similar populations and settings [29–32]. These low-cost, non-invasive practices fit easily into daily routines and align with local religious traditions [33] and wellness approaches [34], making them well suited for mental health strategies in LMICs. Furthermore, their potential effectiveness is enhanced by the well-established importance of integrating cultural factors into mental health strategies in LMICs [33,35–38].

We identified a yoga and compassion meditation program (YCMP) from Brazil (which we refer to as Danucalov et al.’s YCMP) that was originally designed for family caregivers of individuals with Alzheimer’s disease. This program is delivered in-person and is aided by a DVD for home practice, and it has been shown to effectively reduce stress in challenging caregiving situations [39,40]. Given the success and time efficiency of the original YCMP, we tailored the intervention into a culturally relevant and condensed version for mothers of children with ID in Nepal, who face similar stress and caregiving challenges.

## Aims

This study assessed the feasibility of our contextually adapted YCMP, focusing on its practicality and acceptability for mothers of children with ID in Nepal. We explored the participants’ insights regarding our YCMP’s cultural and contextual relevance, perceived benefits, implementation, sustainability, and associated challenges through focus group discussions (FGDs). This feasibility study thus lays the groundwork for future research and intervention development, and it represents an advancement in health promotion by empowering these mothers and thus improving their quality of life.

## Methods

### Consultation with users and adaptations

Understanding the implementation context is essential for developing effective interventions [41]. We previously conducted indepth interviews with mothers of children with ID in province 3 of Nepal [10]. These interviews highlighted the unique challenges faced by these mothers, such as the loss of employment, financial hardship due to caregiving, and the significant role of spiritual practices in both accepting their child with ID and developing coping strategies.

With this contextual knowledge, the first author presented the key components of the original Danucalov et al. (2017) YCMP to this study’s collaborative partners, including board members from the Parents Federation of Person with Intellectual Disability (PFPID), the Down Syndrome Society, the National Federation of Disability Nepal (NFDN), and leaders from local day care centres for children with ID. A discussion meeting with six representatives from these organizations and with two mothers who used the day care centres, which are operated by parents and relatives of children with ID, formed this study‘s user-group.

The group responded positively to the YCMP components and expected the study participants to comprehend it. However, given the mothers’ limited time and their additional household responsibilities, the mothers suggested that we simplify the Danucalov et al. YCMP by condensing the content and reducing the number of in-person follow-up sessions. Additionally, the user group recommended incorporating weekly check-in phone calls to connect with and provide support to the participants, address questions, and clarify any doubts regarding the at-home practice of YCMP. These adaptations aimed to enhance participation, motivation, and adherence.

Further adaptation to Danucalov et al.’s YCMP [39,40] included the development of a take-home package that included weekly tracking logs in a calendar format with yoga positions (*asanas*), notebooks for recording queries, yoga mats for home practice, and a social media group with videos of the yoga asanas and breathing techniques (*pranayama*). The package also included a brochure outlining information about the YCMP and the overall research project. Table 1 provides an overview of these adaptations.

**Table 1.** Adaptations to the yoga and compassion meditation intervention for Nepalese mothers of children with intellectual disabilities.

Component	Original (Danucalov et al. [39,40])	Adaptation for Nepalese mothers	Rationale for adaptation
Yoga body poses (*asanas*)	25-minute sequence: Sukhasana, Vajrasana, Yoga-Mudra, Paschimottanasana, Ardha-Matsyendrasana, Shavasana, Naurkasana, Bhujangasana, Ardha-Shalabhasana, Standing Chakrasana, Vrikasana, Sarvangasana	25-minute sequence: Sukhasana, Vajrasana, Paschimottanasana, Suryanasmaskar, Balasana, Makarasana, Bandakonasana, Janu Sirshasana, Viparit Karni Asana, Malasana, Shavasana	The selection of the adapted poses took advantage of the instructor’s familiarity and expertise, thus facilitating easier learning for the participants. This approach also aimed to improve adherence and alignment with local practice styles.
Breath regulation (*pranayamas*)	25-minute sequence including: Adhama, Pranayama, Bhastrika, Ujjayi, Surya Bhedana, Chandra Bhedana, Nadi Shodhana, Kapalbhati	25-minute sequence including: Pranayama, Kaalbhati, Anulom Vilom, Sitali Pranayama, Bhastrika	Simplified to focus on techniques that participants can easily remember and practice independently. This was intended to enhance the feasibility of the program.
Mindfulness meditation	12.5 minutes	7 minutes	No change was made.
Compassion meditation (*karuna*)	12.5 minutes	7 minutes	No change was made.
Training duration and frequency	Two months, 75 minutes per session, three times per week.	14 weeks (3.5 months), 64 minutes per session, initial training plus one refresher session at 6 weeks after initial training.	Extended duration with reduced live sessions to better fit the participants’ schedules, while the refresher session was intended to enhance retention and reinforce learning.
Live sessions and at-home practice	One live session per week (8 live sessions total), with two additional at-home sessions per week aided by DVD (16 sessions).	Two live sessions (initial and refreshing training), with weekly follow-up phone calls. In addition, there were videos shared via YouTube, a private Facebook group, and Viber, as well as trackable calendars provided with the yoga asanas and additional aids (notebooks, brochures, and yoga mats).	The follow-up through phone calls and the use of interactive digital resources was intended to ensure continuous engagement and support. Trackable calendars and additional materials were designed to facilitate adherence and make practice more accessible and motivating.

### Intervention design and delivery

The total intervention spanned 14 weeks and was conducted at three different locations for the convenience of the participants. After an initial 1.5 hour in-person YCMP training session (including introductions and practice), the participants received their take-home packages. Follow-up about yoga positions, meditation practices, and answering queries then occurred during weekly phone calls, while the dedicated social media group was used for video accessibility and participant engagement. Six weeks after the initial training session, refresher training sessions were conducted in the same three locations. The FGDs among participants were carried out 2 weeks after the refresher training and about 4 months after the initial YCMP sessions.

During the initial and refresher YCMP sessions, a female yoga instructor guided participants through the program’s yoga and meditation components using relatable, real-life examples. For example, to demonstrate the technique of forceful expiration, she asked participants to imagine holding an axe, inhaling deeply, and then forcefully exhaling as if splitting a log. These examples helped the mothers to grasp the concepts of the intervention. The program started with breathing techniques (*pranayama*), followed by body postures (*asanas)* focusing on breath awareness and regulation. Meditational practices included mindfulness and compassion meditation (*karuna*), emphasizing attention to the present moment and the production of compassionate feelings for all living beings, including oneself. The refresher sessions allowed for questions, feedback on home practice, emotional sharing, and collective practice with the breathing techniques, yoga *asanas*, and meditation. At the refresher sessions, an invitation card was extended to the participants to participate in the FGDs.

### Data collection and analysis

#### Participant recruitment

Out of the 51 mothers of children with ID who were invited to participate in the intervention study, 40 agreed and took part in the YCMP intervention. This article focuses exclusively on the findings from FGDs with 24 of these mothers. Participants were recruited face-to-face during the YCMP training sessions, where printed invitations were distributed. One week prior to the FGD sessions, follow-up reminder phone calls were made to confirm attendance and logistical details.

The participants were recruited through daycare centres and local organisations serving families with children with ID in the region. Mothers were invited to participate based on their being primary family caregivers of a child with an ID and their residing in Province 3, Nepal. Purposive sampling was used to ensure diversity in age, socioeconomic background, and caregiving experience. The sample included mothers primarily from low-income socioeconomic backgrounds, with several engaged in informal or daily wage labour. Most participants had limited formal education, and only a few reported holding regular salaried jobs, such as tailoring, shop keeping, or caregiver. Participation was voluntary, and participants were informed that they could withdraw at any time.

#### Data collection

The FGDs were guided by an interview guide focusing on the participants’ thoughts on the acceptance and practicality of the intervention as well as challenges related to the intervention’s implementation. The first author moderated the discussion, thus ensuring a conducive environment for open dialogue. The FGD sessions were meticulously documented through audio recording and notetaking, and the audio files were subsequently transcribed and translated from Nepali to English for data analysis.

#### Sample size and saturation

A total of three FGDs were conducted with 24 mothers in three locations: Dhulikhel (6 participants), Lalitpur (9 participants), and Kathmandu (9 participants). The sample size was guided by the principle of thematic saturation, defined as the point at which no new themes or insights emerge from the data [42]. Saturation was assessed iteratively during data analysis and was observed by the second and third FGDs, when key themes were consistently repeated across groups. This approach is also consistent with Glaser and Strauss’s description of saturation as the point at which no new information emerges and similar patterns are repeatedly observed, providing sufficient depth and breadth for small, focused qualitative studies [43].

#### Data analysis

The constant comparative method, which is commonly used to analyse qualitative data from multiple participants [43,44], was initially applied to process the data [43]. This approach involved comparing data within and across the FGDs to identify emerging themes. Following this, a thematic analysis was conducted using a combined deductive and inductive approach. The deductive aspect of analysis was guided by Bowen et al.’s feasibility framework [45], focusing on components such as acceptability and practicality, which are crucial dimensions for assessing the feasibility of any intervention. However, the analysis also allowed for the emergence of inductively derived, data-driven themes that were unique to the intervention context. This process identified both predefined key themes based on Bowen et al.’s framework and novel subthemes that provided additional insights into the feasibility of the intervention.

#### Reflexivity

The lead facilitator was trained in qualitative research and had prior experience in community-based health services for persons with disabilities and in working with organisations for persons with disabilities in Nepal. While there were no close personal relationships with participants, several meetings held before, during, and after the intervention helped build familiarity and trust between the research team and participants. This ongoing engagement appeared to foster a sense of openness and comfort, encouraging participants to speak candidly during the group discussions. At the same time, the research team remained reflexive about how this familiarity, along with their own positionalities as researchers invested in the intervention’s success, might influence participant responses. To mitigate potential bias, reflexive notes were kept throughout data collection and analysis, and findings were discussed collaboratively within a multidisciplinary team. This allowed for critical reflection on interpretation and helped ensure a balanced and credible analysis.

## Results

**Figure 1.**
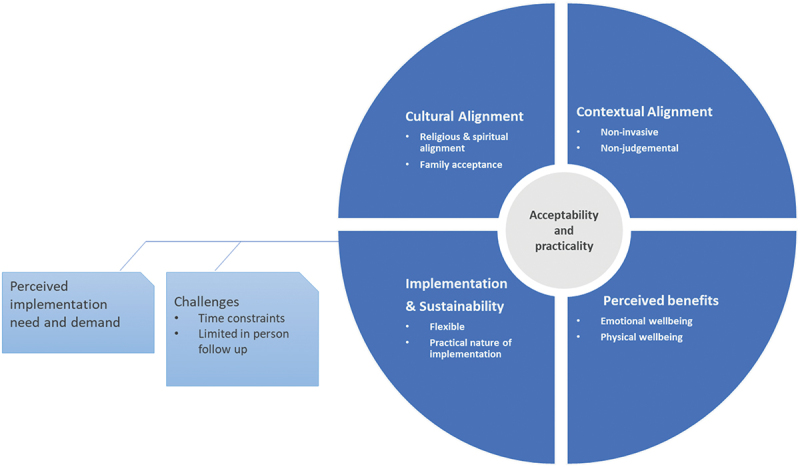
Findings on the feasibility of the YCMP intervention for mothers of children with intellectual disability in Nepal, guided by Bowen et al. [45] feasibility framework, highlighting acceptability and practicality as key dimensions.

Our findings highlight acceptability and practicality as key dimensions using Bowen et al. [45] framework for assessing the feasibility of the YCMP. These two dimensions are crucial in determining how well an intervention is received and its feasibility in real-world implementation. As shown in Figure 1, there were several sub-dimensions of both the feasibility and practicality of the YCMP, including cultural and contextual alignment, perceived benefits, implementation processes, and sustainability considerations. We also identified challenges related to implementation and sustainability, which are presented in the figure as a distinct domain to highlight the barriers that were faced during the intervention’s implementation. The following sections delve deeper into these findings, providing a comprehensive assessment of the YCMP’s overall feasibility.

All participant quotes presented have been anonymised using randomly assigned pseudonyms (e.g. ‘Sabina,’ ‘Sita’) that do not correspond to participants’ real names. These were selected to enhance readability while ensuring confidentiality. Any identifying details shared during the discussions were removed or altered during transcription to protect participant privacy.

### Perceived benefits: physical and emotional wellbeing

Participants consistently reported that the YCMP improved both their physical and emotional wellbeing, describing these in interconnected ways such as feeling more energetic, relaxed, and able to manage stress, with yoga and meditation contributing to reduced physical tension and enhanced emotional regulation. For example, Sita shared how her chronic upper back pain had improved through YCMP practice, noting, ‘*I feel yoga has helped me with the pain and the [underlying] problem, and yoga is a better option than taking medication.’* Other participants echoed similar physical benefits, including pain relief and increased energy. For instance, Junu remarked that her morning yoga practice helped boost her energy levels throughout the day.

Emotionally, participants spoke of feeling calmer, more composed, and better able to manage frustration, particularly in challenging caregiving situations. Bidhya described how she previously struggled with anger toward her child but now felt more accepting and able to control her emotions: ‘*I have realized I shouldn’t yell or be angry … now I can control my anger and frustration better than before*.’ Radha also emphasized a sense of inner peace and clarity: ‘*It clears my mind and makes me feel blissful … I started teaching it to my daughter as well*.’ Collectively, these reflections illustrate how the YCMP was perceived to promote physical vitality and emotional resilience in the daily lives of mothers raising children with ID.

### Cultural alignment: fit with spiritual beliefs and family acceptance

Participants shared that the YCMP aligned well with their spiritual, religious, cultural, and daily practices. They described how the program’s consistency with Hindu and Buddhist traditions – particularly through the familiar practices of yoga and meditation – made it easy to integrate into their daily routines. These practices were already well known and widely accepted in their households and communities and were often promoted by religious leaders on TV, which made the YCMP feel familiar and appropriate for them. This alignment with spiritual traditions was further supported by family encouragement. Sabina, a mother from Lalitpur, shared why her family supported her participation in the YCMP: *‘My family would not have liked it or allowed it if I left home to learn dancing or music or aerobics … but since everyone knows that yoga and meditation have benefits and have gained popularity through TV shows as well, my family members encouraged me to practice it … I found it very beneficial too.’*

Participants also noted that their familiarity with yoga and meditation, along with a growing interest in and inclination towards alternative medicines and wellness techniques, enhanced the program’s relevance and acceptability for them.

Several participants shared that they preferred the YCMP over other wellness programs, such as dance or aerobics, because it was more culturally acceptable and better suited to their needs. The program’s adaptability and non-intrusive nature made it a feasible and sustainable option for improving their wellbeing.

### Contextual alignment: a non-invasive and non-judgmental approach

In addition to its cultural fit, the participants appreciated the non-invasive and non-judgmental nature of the program. Bidhya from Kathmandu said, *‘I prefer doing this [yoga and meditation] for my wellbeing rather than going to a doctor … which costs money.’* To this, Ramila added, *‘If I go and seek help from medical professionals for my tensions [referring to mental health issues] … even my own family members see me as a crazy/mad person … so, I [appreciate] trying yoga and meditation first to manage my health.’* Other participants also noted a preference for non-invasive, conservative treatments, feeling more comfortable with such interventions for managing their wellbeing than seeking professional help.

#### Implementation and sustainability: flexible and practical nature of implementation

Participants consistently highlighted the intervention’s flexibility, noting how it accommodated their individual preferences and fit easily into their daily routines. Sita, for example, seamlessly incorporated the YCMP practices into her day while cooking or relaxing in the evenings on her terrace. She said, *‘Practicing yoga and meditation has become a habit for me now … I have realized that it is good … my belly fat is gone … I do it when I am in the kitchen, cooking, and in the evenings … it helped me with the irritation too*.’ Despite her initial scepticism about time constraints, Sita appreciated that the practices required no special equipment or designated time, making them accessible: *‘The fact that we can practice it at home and at any time of the day makes it easy for us to incorporate in our lives. I practice it whenever I am free.’*

Hasina also noted how easily she incorporated the YCMP into her morning routine with minimal disruption: *‘Usually, we all have a habit of waking up early in the morning … So, it is not a big deal. It was easy to practice and incorporate in our daily lives.’* This flexibility and the option for at-home practice were widely viewed as enhancing the practicality and potential sustainability of the intervention, allowing mothers to incorporate the YCMP practices into their everyday lives without adding extra burden.

#### Adaptability of tailored take-home packages

The YCMP intervention was customized to meet the diverse needs and preferences of participating mothers through tailored take-home packages, which included instructional videos and calendars. These resources were designed to accommodate participants’ varying levels of access to technology and different usage preferences. Most mothers favoured videos for their convenience in demonstrating yoga asanas and their ease of use. Participants such as Bharati, Radha, and Sita found the videos handy for reviewing the asanas at their convenience. Bharati said, *‘The videos were very handy … [for] check[ing] if I forgot or missed any asanas.’*

Conversely, some mothers preferred the calendars due to a lack of access to smartphones or time constraints. Romita, for instance, found the calendars more useful and convenient: *‘I never have my phone with me; my children are always using it. I have the calendar placed on a table, and I find it handy to go through the pictures and information on the calendar.’*

All of the mothers appreciated the yoga mat that was provided, finding it useful and motivating for their yoga practice. Sita expressed, *‘I really love the yoga mat; it is so useful, and easy to carry everywhere. It motivates me to do yoga and, in a way, reminds me to practice.’* Minimal use was reported for brochures and notebooks, indicating a lower preference for these materials.

#### Perceived needs and demands for implementing the YCMP

According to Bowen et al. [45] feasibility framework, demand refers to participants’ interests, intentions to use, and perceived needs for an intervention. The framework assesses participants’ likelihood of engaging with and valuing the intervention, thus helping to determine its feasibility and potential success. For the mothers in this study, the YCMP was a novel intervention focused on their wellbeing. While some participants were initially sceptical about its impact on their stress levels, their doubts diminished as they experienced benefits such as better emotional regulation. As their appreciation for the program grew, so did their interest and intention to continue using it.

Ramila highlighted the uniqueness of the YCMP’s focus on mothers’ stress and wellbeing: *‘All the other programs and workshops we heard about or participated in are always targeted to our children. We always focus on our children and ignore our own needs. But this is the first program for us – mothers like us who face many challenges and stressful situations. I had never thought about how important it was to manage my stress and the difference it makes in our lives, our children’s lives, and our family members’ lives. I really think we need this kind of program to help manage everyday stresses and anxiety and achieve good health.’*

Ramila’s experience underscores the YCMP’s perceived value in addressing the specific challenges faced by mothers of children with ID. Her reflection on her newfound awareness of the importance of stress management and self-care, along with similar feedback from other participants, highlights the high demand and relevance of interventions like the YCMP.

#### Challenges: time constraints and follow-up limitations

Participants in the YCMP faced challenges in practicing yoga and meditation regularly, largely due to the demands of their household responsibilities and their professional commitments. These challenges were further compounded by structural issues such as poverty, limited external support, and the low social status of women in Nepal, factors that all of the mothers mentioned as prevalent in their lives.

Many mothers, particularly those with demanding jobs outside the home and those with younger children, found it difficult to engage consistently with YCMP practice. Meera, for example, expressed her desire to practice daily but noted that her busy days left her with only a few opportunities to practice each week: *‘I really feel good when I practice YCMP; however, I am busy with household chores on some days, and then I cannot make time for it. Therefore, I cannot practice it every day. However, I practice it a few days, sometimes two days a week and other times four. It varies.’*

A lack of external support for caregiving also limited the ability of many mothers to participate fully in the intervention. Nabina shared that when her children were sick, the demands on her time made it impossible to practice: *‘Sometimes my children demand so much of my time, especially when they are sick and unwell, that I cannot practice it at all. And there is no one I can rely on.’* Similarly, Lata described the difficulty of maintaining focus during YCMP practice due to the constant noise and activity caused by her children: *‘It is impossible to practice YCMP. My children always make noises and run around, which makes it hard for me to practice it and meditate well.’*

Although the weekly follow-up calls provided ongoing support and helped integrate YCMP practices into their daily lives, some mothers expressed a need for more frequent group sessions and follow-up sessions to maintain their motivation and effectively incorporate the YCMP practices into their daily routines. However, the ability to attend frequent sessions was also constrained for many participants. Some mothers suggested monthly sessions for knowledge refreshment and motivation, while others recommended weekly sessions, highlighting varying levels of support needed to sustain participation. This also underscores the challenges faced by women who have fewer resources, less time, and less external support for their caregiving roles.

## Discussion

This study explored the feasibility and acceptability of our adapted YCMP through FGDs with mothers of children with ID in Nepal. Overall, the intervention consistently emerged as both acceptable and practical for participants. These key indicators of feasibility were reflected in the perceived benefits of the program, its alignment with cultural and contextual norms, its ease of implementation, and its ability to address a clear need among caregivers of children with ID. Our findings suggest that culturally relevant and practical interventions like the YCMP may play a critical role in supporting the physical and emotional wellbeing of caregivers, even in settings marked by significant contextual constraints and challenges.

### Cultural alignment and feasibility in health-promoting interventions

Existing research emphasizes the importance of cultural alignment for successful health interventions [36,37], and studies consistently show that interventions tailored to religious and cultural norms are more effective [33,35,46]. Our findings highlight that the feasibility of the YCMP was closely tied to its cultural fit.

Participants responded positively to the YCMP not only for its perceived stress-reducing benefits but also due to its congruence with deeply rooted spiritual practices such as mindfulness and meditation. Given the strong Hindu and Buddhist influences in Nepalese culture [47], the embeddedness of familiar religious and cultural elements likely enhanced the program’s resonance with participants’ values and traditions [18]. This cultural compatibility made it easier for participants to incorporate the program into their daily lives, suggesting that adherence to spiritual traditions contributed significantly to the intervention’s acceptability and success. Health-promoting interventions that align with local cultural frameworks are more likely to be embraced, thus empowering and motivating participants to make positive choices for their health and wellbeing [48,49].

Furthermore, family support played a crucial role in sustaining participants’ engagement with the YCMP. In Nepal’s patriarchal society, family acceptance, especially when linked to culturally familiar practices, strongly influences women’s ability to participate in health-promoting activities [50]. Family approval not only facilitated [48] but also encouraged consistent YCMP practice at home. These findings underscore the importance of cultural sensitivity when designing interventions for populations with distinct cultural backgrounds. In contexts like Nepal, where mental healthcare remains stigmatized and under-resourced [32,37,49], culturally familiar practices like yoga and meditation offer a ‘safe’, accessible, and socially acceptable alternative [51,52].

### Perceived benefits and the complex nature of caregiving

The perceived benefits of the YCMP, spanning emotional regulation, relief from physical pain/discomfort, and the adoption of proactive coping strategies, reflect the multifaceted burden faced by mothers of children with ID. Rather than viewing these as isolated outcomes, they should be understood within a context where emotional distress is often somatically expressed and mental health services remain stigmatised and inaccessible [53,54]. Evidence from mindfulness-based interventions indicates potential neurobiological pathways, such as enhanced mood-regulating neurotransmitters, which may explain these perceived gains [34,55]. Importantly, the YCMP offered a form of culturally acceptable, non-clinical support that helped women reframe caregiving from reactive to preventive self-care, challenging entrenched gender norms of self-sacrifice common in Nepal and similar LMIC contexts [9,47,50].

By creating temporary respite and encouraging self-compassion, the program addressed both the physical and emotional labour of caregiving, aligning with feminist perspectives, on agency and wellbeing in restrictive social contexts [5,9,48–50]. These findings suggest that the family caregiver-focused interventions in LMICs can serve as both stress-management tools and subtle acts of resistance against norms that deprioritize women’s health [6,10,56].

### Contextual barriers: socioeconomic factors and feasibility

Our findings highlight broader contextual barriers influenced by the socioeconomic challenges faced by family caregivers, particularly mothers of children with ID, in LMICs. These women often bear the double burden of unpaid caregiving and household responsibilities, and this is exacerbated by limited external support, poverty, and gender inequities [10,13,14,57]. Together, these factors contribute to the socioeconomic vulnerabilities they experience. Although addressing socioeconomic vulnerabilities was not the primary aim of our study, these factors critically impact the feasibility and implementation of the YCMP [46]. In contexts like Nepal, gender inequities limit access to healthcare, employment, and other essential resources. Interventions must therefore consider these realities to ensure their feasibility and sustainability.

Several mothers in our study struggled to engage regularly with the YCMP due to limited access to respite care, limited external support, and the overwhelming demands of caregiving. These challenges contribute to ‘time poverty’, which often results in self-neglect, poor health choices and outcomes, limited access to healthcare, and increased stress [58]. These barriers create substantial obstacles to the consistent implementation of health interventions like the YCMP, underscoring the importance of considering gender and socioeconomic disparities in the design of such programs.

Our YCMP was specifically designed to improve the wellbeing of the mothers, rather than their children, and this focus was highly valued by the participants. One key highlight of this study was the importance of understanding and addressing contextual needs when designing and implementing health interventions. Despite existing disability policies in Nepal, mothers and families of children with ID often lack adequate support because the healthcare system primarily focuses on the treatment and needs of the children, thus neglecting the broader psychological needs of these mothers [59]. This gap highlights the potential role of the YCMP in providing immediate stress relief and promoting the long-term wellbeing of mothers while also informing future policy changes.

### Implementation and sustainability: flexible and practical

The flexible nature of the YCMP enhanced its practicality for the participants, many of whom faced time constraints due to constant caregiving responsibilities. These findings reinforce the importance of flexibility in intervention delivery, especially in low-resource contexts where caregiving duties often leave little time for structured health programming [60]. Participants reported that adaptable schedules were essential to their ability to engage with the YCMP practices, and a key insight from this study is that public health interventions are more likely to be feasible when they seamlessly fit into participant’s existing routines [57]. Thus, a resource-efficient design that incorporates peer support is vital to promoting self-sufficiency and community capacity building and thus empowering women’s healthcare [61]. This also enhances the sustainability and long-term impact of the YCMP.

The YCMP’s design emphasized self-sufficiency by equipping caregivers with tools to enhance their wellbeing without heavy reliance on external resources. This is particularly crucial in patriarchal societies, where women’s health needs are often deprioritized. In these contexts, a flexible and practical approach can expand women’s capacity to make autonomous decisions, thus allowing them to reclaim some control over their health and wellbeing within restrictive social contexts [62]. By addressing contextual barriers, the YCMP can also help strengthen social networks and support systems, thus creating a more inclusive environment for these mothers [63,64].

Moreover, the YCMP facilitated peer support, thus enabling participants to share their experiences, navigate challenges, and gain acceptance [65–67]. This collaborative environment not only fosters engagement without the burden of stigma but also increases the likelihood of successful implementation and sustainability [65,68]. As participants develop stronger connections and a shared commitment to their wellbeing, the program’s long-term success is further supported.

### Implementation and sustainability challenges

While our YCMP study demonstrated feasibility, it also revealed implementation challenges, particularly related to time constraints. These were closely linked to structural and gender-based barriers, such as entrenched expectations that women carry the primary responsibility for both unpaid caregiving and household work, leaving little discretionary time for self-care. In many cases, participants also had limited autonomy to prioritise their own health needs, as decisions about time use and mobility were influenced by other family members. Such constraints, widely documented in studies on time poverty and the double burden of caregiving in low-resource settings [14,46], impeded the consistent uptake of health-promoting interventions.

To address these implementation challenges, future iterations should consider flexible, scalable interventions that can be easily adapted to varying circumstances and delivered by different stakeholders, such as day care centres, peer groups, and community health workers. Incorporating digital platforms, peer support groups, and extended follow-ups could enhance engagement and motivation, as evidenced by successful health interventions and peer support group studies [19,67].

### Limitations and transferability

This study has several limitations that should be acknowledged. First, the sample was limited to 24 mothers of children with ID who participated in FGDs in one district of Province 3, Nepal. While their perspectives provide valuable insights into the feasibility and cultural acceptability of the YCMP, transferability to caregivers in other regions may be limited due to differences in cultural practices and caregiving contexts. Second, the study relied on self-reported data collected through FGDs, which may have been influenced by social desirability bias or reluctance to share sensitive views in a group setting. The researcher’s familiarity with some participants may have fostered openness but could also have subtly shaped responses or interpretations.

Although data saturation was sought, the perspectives of non-participating mothers were not included, which may have narrowed the range of experiences captured. As a feasibility study, the aim was not to assess intervention outcomes or effectiveness, so conclusions about impact should be drawn cautiously. Moreover, the exclusive use of FGDs with participating mothers, without triangulation from other stakeholders, may have limited the breadth of perspectives.

Despite these limitations, the study offers valuable insights about the cultural acceptability and practicality of implementing a caregiver-focused intervention in a low-resource Nepali setting. In line with qualitative research principles, transferability relies on the richness of contextual detail provided, enabling readers to assess the relevance of the findings to their own settings [69]. At the same time, the results hold broader conceptual value for informing the design of caregiver support interventions in similar contexts.

## Conclusion: implications for future research

This study demonstrates the acceptability and practicality of the YCMP among mother caregivers of children with ID in Nepal, ensuring the feasibility of our intervention. The YCMP’s acceptability and practicality were closely tied to its cultural alignment, adaptation to the specific context, flexibility, and resource-efficiency, all of which are crucial factors for successful implementation.

By promoting a culturally sensitive, non-invasive approach and addressing some key contextual barriers, the YCMP holds potential for enhancing equitable access to care and support for mothers of children with ID in Nepal. Integrating such interventions into the public health system is an important step, but it is essential to consider broader strategies for health promotion in such marginalized populations in order to ensure that all individuals benefit from quality healthcare.

Future research should focus on scaling up the YCMP, expanding its reach to a larger population while evaluating and ensuring cost-effectiveness and long-term impact. Rigorous quantitative studies, such as randomized controlled trials, can provide stronger evidence of the program’s impact and validate its applicability in resource-limited settings. Including larger and more diverse populations would also help assess the program’s scalability across different regions and demographics, thus allowing for necessary adaptations to diverse cultural contexts.

Furthermore, a broader implication for public health interventions in LMICs is the necessity of designing programs that are not only culturally and contextually aligned, but that are also sustainable. Sustainability can be achieved by addressing logistical barriers such as infrastructure limitations, workforce constraints (lack of trained professionals), and access to resources. By considering these factors, interventions can have greater long-term impacts.
